# Relative Validity of a Short Food Frequency Questionnaire for Assessing Nutrient Intake versus Three-day Weighed Diet Records in Middle-aged Japanese

**DOI:** 10.2188/jea.15.135

**Published:** 2005-08-30

**Authors:** Yuko Tokudome, Chiho Goto, Nahomi Imaeda, Takako Hasegawa, Rieko Kato, Kaoru Hirose, Kazuo Tajima, Shinkan Tokudome

**Affiliations:** 1 Department of Health and Nutrition, School of Health and Human Life, Nagoya-bunri University.; 2 Nagoya Women’s University.; 3 Nagoya-bunri College.; 4 Department of Health Promotion and Preventive Medicine, Nagoya City University Graduate School of Medical Sciences.; 5 Division of Epidemiology and Prevention, Aichi Cancer Center Research Institute.

**Keywords:** Energy Intake, Questionnaire, Micronutrients, Diet Records, Validity

## Abstract

BACKGROUND: Validation studies on brief food frequency questionnaires (FFQs) for measuring consumption of macro- and micro-nutrients for the general populace are not fully executed in Japan.

METHODS: Two hundred and two middle-aged Japanese (73 males and 129 females) in Aich Prefecture, Japan completed an FFQ and 3day-weighed diet records (3d-WDRs) in February 2004. We compared intakes of energy and 26 nutrients computed with the FFQ against those with the 3d-WDRs as a reference.

RESULTS: Mean daily intakes of selected nutrients determined with the FFQ were generally less than those with 3d-WDRs. The ratios assessed with the FFQ vs. 3d-WDRs (minimum - median - maximum) were distributed from 0.57 - 0.79 - 1.09 for males, and 0.61 - 0.86 - 1.04 for females. De-attenuated, log-transformed and energy-adjusted Pearson’s correlation coefficients between intakes of selected nutrients quantified with both devices were distributed from 0.12 - 0.45 - 0.86 and energy-adjusted Spearman’s rank correlation coefficients were from 0.13 - 0.35 - 0.76, for males. The respective values for females were 0.10 - 0.38 - 0.66, and 0.11 - 0.34 - 0.47. Median percentages for exact agreement, agreement within adjacent categories, and disagreement according to quartile classification of the energy-adjusted nutrient intakes measured with both methods were 33, 74, and 5 for males, and 35, 76, and 7 for females, respectively.

CONCLUSION: Satisfactorily high relative validity indices of most nutrient intakes computed with the FFQ were attained against those with the 3d-WDRs. The questionnaire therefore appears applicable for categorizing individuals according to consumption of energy and selected nutrients in dietary studies of middle-aged Japanese.

It is well known that the leading causes of death are now chronic diseases such as cancer, cerebrovascular problems and heart disease in developed countries, including Japan.^1^ They are related to daily lifestyle, including dietary habit, alcohol drinking, smoking, physical exercise, and factors for stress. Because dietary habit, in particular, appears to play a major role in their pathogenesis, batteries of tests to assess intake of foods/nutrients, including fats/fatty acids, antioxidants and dietary fibers, are needed for epidemiologic studies.

There are several tools available, including diet records (DRs)/weighed diet records (WDRs), 24-hour recall, food frequency questionnaires (FFQs), and duplicate methods.^2^^-^^4^ Among these, the FFQ is most often employed to evaluate associations between long-term food intake and health/disease. We earlier developed a data-based semi-quantitative food frequency questionnaire (SQFFQ) using multiple regression analysis (MRA) as well as contribution analysis on the basis of WDRs,^5^ and conducted a calibration/validation and reproducibility study, as detailed elsewhere.^6^^,^^7^ However, the SQFFQ was primarily designed for the JADE (Japanese Dietitians’ Epidemiologic) Study. We recently evolved a self-administered brief FFQ according to MRA as described elsewhere^8^ for epidemiologic studies on lifestyle-related diseases of the middle-aged Japanese general populace.

In the present investigation, we carried out a relative validity study of intake of energy and 26 macro- and micro-nutrients measured with our FFQ versus reference values with three-day WDRs (3d-WDRs).

## METHODS

### Subjects

We recruited 222 (83 males and 139 females) middle-aged volunteers (30 - 70 years of age) who were attending physical exercise classes in communities, or were parents of college students in Aichi prefecture, central Japan. Twenty individuals were excluded from this study because eight persons were under 30 or over 70 years old, eight had not complied with the research regimen and four whose responses for energy lay beyond 3 standard deviations (SDs) from the mean measured with the FFQ. Finally, 202 participants (73 males and 129 females) were thus included in the present analysis.

### FFQ and 3d-WDRs

In February 2004, we first administered the FFQ to the subjects by mail. The questionnaire inquired about habitual dietary intake during the previous one year for 47 foods/recipes and frequency in eight categories: never or seldom, 1-3 times/month, 1-2 times/week, 3-4 times/week, 5-6 times/week, once/day, twice/day and more than three times/day. For staple foods, including rice, noodle and bread, the portion size/serving size was requested. Approximately one week later, we administered the 3d-WDRs (two week-days and one weekend) and a disposable camera to photograph foods when eating out or take-out. Diet records not completed were checked and verified by research dietitians.

### Nutrients selected

We earlier developed a brief FFQ for energy and 26 macro- and micro-nutrients, including protein, fat [saturated fatty acids (SFAs), mono-unsaturated fatty acids (MUFAs), poly-unsaturated fatty acids (PUFAs), n-6 PUFAs, n-3 PUFAs, n-3 HUFAs (highly-unsaturated fatty acids) and cholesterol], carbohydrate, total dietary fiber (TDF) (soluble and insoluble), minerals (calcium and iron) and vitamins (carotene, and vitamins A, C, D and E),^8^ and added 6 nutrients of interest, including carbohydrate energy%, protein energy %, fat energy %, vitamin B_1_, vitamin B_2_ and folate.

### Calculation of intake of nutrients

We computed the average daily consumption of energy and selected nutrients using information from the FFQ and lifestyle questionnaire, including consumption of alcohol. According to the regression analysis, selected nutrients were adopted as dependent parameters and foods/food groups consumed, intake frequency, portion size (in grams) from our database,^5^^,^^8^ or typical/standard values from the literature, nutrient contents per 100 grams of foods/food groups listed in the respective composition tables or of the model recipes were assumed to be independent variables.^9^^-^^13^ With the WDRs, we calculated mean daily intakes of selected nutrients by multiplying the consumption of foods/food groups (in grams) and nutrient contents per 100 grams of foods as listed in the composition tables or model recipes.

### Validation

First, we compared mean daily intakes of energy and 26 selected nutrients gauged with the FFQ against those with the 3d-WDRs. Differences in means and ratios were computed with the FFQ vs. 3d-WDRs values, and examined by t-test using Excel^®^ and the SPSS^®^-10.0 software package.

Second, we calculated crude Pearson’s correlation coefficients (CCs), log-transformed Pearson’s CCs, log-transformed and energy-adjusted Pearson’s CCs, and de-attenuated, log-transformed and energy-adjusted Pearson’s CCs between intakes of selected nutrients based on the FFQ and 3d-WDRs. Energy adjustment was executed using regression models.^14^ De-attenuated Pearson’s CCs were computed by partitioning within- and inter-individual variations by one way of analysis of variance according to the formula described elsewhere.^3^^,^^15^^-^^17^ Crude Spearman’s rank CCs and energy-adjusted Spearman’s rank CCs were also calculated.^18^^,^^19^ Statistical significance was verified with the 95% confidence interval.

Third, after categorizing daily intakes of nutrients quantified with the FFQ and 3d-WDRs into quartiles, we computed percentages of exact agreement, agreement within adjacent categories, and disagreement.

### Ethical issues

Our study protocol was reviewed and approved by the Internal Review Board at Nagoya City University Graduate School of Medical Sciences. Written informed consent was obtained from each participant.

## RESULTS

### Profile of study subjects

The mean ages ± standard deviations (SDs) (minimum - maximum) were 51.7 years ± 8.9 (30 - 68) for males, and 49.6 years ± 8.8 (30 - 68) for females. The values for height, weight and body mass index (kg/m^2^) were 169.6 cm ± 6.6 (145.3 - 187.0 ), 65.5 kg ± 8.7 (53.0 - 93.0), and 22.7 ± 2.3 (18.0 - 28.1) for males, and 155.8 cm ± 5.2 (142.1 - 171.0 ), 53.1 kg ± 6.6 (38.5 - 83.0 ), and 21.7 ± 2.2 (16.9 - 28.4) for females, respectively.

### Intake of nutrients

The intakes of energy and macro- and micro-nutrients gauged with the FFQ were generally lower than those with 3d-WDRs (Table 1). The ratios of nutrient consumption measured with the FFQ vs. 3d-WDRs (minimum- median- maximum) were distributed from 0.57 - 0.79 - 1.09 for males and 0.61 - 0.86 -1.04 for females.

**Table 1.** Comparison of daily intakes of energy and 26 nutrients measured with three-day weighed diet records vs. food frequency questionnaire.

Nutrient	Male (n=73)							Female (n=129)					
	
		3d-WDRs		FFQ		Ratio of FFQ to 3d-WDRs	p	3d-WDRs		FFQ		Ratio of FFQ to 3d-WDRs	p
			
		Mean	SD	Mean	SD			Mean	SD	Mean	SD		
Energy	[kcal]	2342	469	1987	268	0.85	***	1924	332	1639	186	0.85	***
Protein	[ g ]	88.4	22.1	60.8	10.2	0.69	***	74.5	16.3	55.2	7.8	0.74	***
Fat	[ g ]	66.1	22.6	47.1	11.9	0.71	***	59.2	16.5	48.4	9.6	0.82	***
Carbohydrate	[ g ]	312.7	57.7	293.0	51.7	0.94	*	264.5	50.0	226.6	36.1	0.86	***

Protein energy%^†^	[ % ]	15.1	2.0	12.3	1.4	0.81	***	15.5	2.0	13.5	1.5	0.87	***
Fat energy%^†^	[ % ]	25.1	5.4	21.4	4.6	0.85	***	27.5	5.1	26.7	4.9	0.97	
Carbohydrate energy%^†^	[ % ]	53.9	6.2	58.8	4.6	1.09	***	55.2	6.1	55.2	5.0	1.00	

Saturated fatty acids	[ g ]	16.6	6.6	11.3	2.0	0.68	***	16.0	5.5	12.4	2.5	0.78	***
Monounsaturated fatty acids	[ g ]	23.1	9.3	17.5	4.4	0.76	***	19.8	6.2	16.9	3.4	0.85	***
Polyunsaturated fatty acids	[ g ]	16.4	5.3	14.1	3.2	0.86	**	14.0	4.1	13.5	2.9	0.97	
n-6 Polyunsaturated fatty acids	[ g ]	12.8	4.5	11.8	2.7	0.92	**	11.0	3.4	11.5	2.6	1.04	
n-3 Polyunsaturated fatty acids	[ g ]	3.3	1.2	2.3	0.5	0.70	***	2.8	1.1	2.2	0.5	0.80	***
n-3 Highly-unsaturated fatty acids	[ g ]	1.1	0.9	0.7	0.3	0.66	***	0.9	0.7	0.7	0.2	0.78	
Cholesterol	[mg]	424	176	274	64	0.65	***	345	132	264	64	0.76	***

Iron	[mg]	9.8	2.4	7.7	1.9	0.79	***	8.9	2.7	7.7	1.6	0.86	***
Calcium	[mg]	592	186	508	129	0.86	*	609	231	566	144	0.93	

Carotene	[*μ*g]	4244	1840	3229	1285	0.76	*	4241	2103	3550	1131	0.84	***
Vitamin A	[*μ*gRE]	989	478	1052	384	1.06		1067	832	1052	422	0.99	
Vitamin D	[*μ*g]	9.4	5.4	7.4	3.4	0.79	**	8.0	5.9	7.2	2.6	0.91	
Vitamin E	[mg*α*-TE]	10.1	3.3	8.6	2.1	0.85	**	9.4	3.0	8.6	1.8	0.92	*
Vitamin B_1_	[mg]	1.18	0.4	0.69	0.08	0.58	***	1.04	0.30	0.70	0.10	0.65	***
Vitamin B_2_	[mg]	1.48	0.44	1.12	0.21	0.76	**	1.38	0.43	1.20	0.20	0.89	***
Folate	[*μ*g]	417	148	357	109	0.86	**	409	164	384	93	0.94	
Vitamin C	[mg]	123	57	103	34	0.84		136	69	122	34	0.90	

Soluble dietary fiber	[ g ]	3.7	1.2	2.1	0.6	0.57	***	2.4	0.7	2.3	0.5	0.61	***
Insoluble dietary fiber	[ g ]	12.1	3.2	8.0	2.2	0.66	***	12.0	3.7	9.0	1.9	0.75	***
Total dietary fiber	[ g ]	16.6	4.4	11.4	3.1	0.69	***	16.6	5.1	12.4	2.7	0.75	***

Median						0.79						0.86	
Average						0.79						0.85	

*: p<0.05, **: p<0.01, ***: p<0.001.

† : Percentage of energy from protein, fat or carbohydrate to total energy.

3d-WDRs: 3-day weighed diet records, FFQ: food frequency questionnaire, SD: standard deviation.

De-attenuated, log-transformed and energy-adjusted Pearson’s CCs between intakes of nutrients quantified with the FFQ and 3d-WDRs were distributed from 0.12 (n-6 PUFAs) - 0.45 (vitamin C) - 0.86 (carbohydrate and carbohydrate energy %) for males (Table 2), and energy-adjusted Spearman’s rank CCs were distributed from 0.13 (n-6 PUFAs) - 0.35 (protein energy % and vitamin D) - 0.76 (carbohydrate energy %).

**Table 2.** Pearson’s and Spearman’s rank correlation coefficients (CCs) between intakes of energy and 26 nutrients measured with three-day weighed diet records and food frequency questionnaire for males.

Nutrient	Pearson’s CCs*							Spearman’s rank CCs*	
	
	Crude	Log- transformed^†^	Log- transformed and energy- adjusted^‡^	*σ*^2^w/*σ*^2^b^§^	De-attenuated, log-transformed and energy-adjusted^|^ (95% CI)			Crude	Energy-adjusted
Energy	0.41	0.40		1.4	0.49	(0.29 - 0.65)	^¶^	0.36	
Protein	0.36	0.32	0.42	1.3	0.50	(0.25 - 0.71)		0.22	0.35
Fat	0.53	0.48	0.52	1.2	0.62	(0.39 - 0.80)		0.38	0.49
Carbohydrate	0.54	0.55	0.73	1.1	0.86	(0.71 - 0.96)		0.57	0.73
						-			
Protein energy%	0.45	0.45	0.42	1.3	0.51	(0.26 - 0.72)		0.38	0.35
Fat energy%	0.55	0.56	0.51	1.2	0.61	(0.38 - 0.79)		0.49	0.50
Carbohydrate energy%	0.68	0.70	0.74	1.1	0.86	(0.71 - 0.97)		0.68	0.76
						-			
Saturated fatty acids	0.50	0.43	0.55	1.0	0.64	(0.48 - 0.90)		0.35	0.52
Monounsaturated fatty acids	0.52	0.44	0.37	1.2	0.43	(0.15 - 0.55)		0.12	0.32
Polyunsaturated fatty acids	0.35	0.31	0.34	1.9	0.44	(0.14 - 0.61)		0.05	0.33
n-6 Polyunsaturated fatty acids	0.20	0.21	0.10	1.6	0.12	(-0.11 - 0.34)		0.20	0.13
n-3 Polyunsaturated fatty acids	0.35	0.36	0.41	2.5	0.55	(0.37 - 0.69)		0.37	0.37
n-3 Highly-unsaturated fatty acids	0.14	0.31	0.28	2.1	0.36	(0.14 - 0.55)		0.28	0.23
Cholesterol	0.35	0.25	0.10	2.1	0.13	(-0.16 - 0.38)		0.15	0.15

Calcium	0.32	0.34	0.42	1.0	0.49	(0.25 - 0.69)		0.38	0.43
Iron	0.25	0.26	0.49	1.2	0.58	(0.35 - 0.76)		0.21	0.50

Carotene	0.19	0.23	0.29	2.18	0.39	(0.09 - 0.65)		0.18	0.28
Vitamin A	0.18	0.16	0.21	2.25	0.27	(-0.03 - 0.55)		0.10	0.19
Vitamin D	0.34	0.40	0.45	3.21	0.65	(0.36 - 0.89)		0.33	0.35
Vitamin E	0.25	0.21	0.25	1.83	0.31	(0.02 - 0.57)		0.16	0.27
Vitamin B_1_	0.31	0.25	0.21	1.73	0.26	(-0.03 - 0.52)		0.19	0.19
Vitamin B_2_	0.35	0.31	0.48	1.11	0.57	(0.36 - 0.77)		0.34	0.53
Folate	0.12	0.17	0.33	0.72	0.36	(0.12 - 0.58)		0.21	0.41
Vitamin C	0.27	0.27	0.40	0.94	0.45	(0.21 - 0.66)		0.24	0.52
						-			
Soluble dietary fiber	0.04	0.07	0.21	1.38	0.25	(-0.03 - 0.50)		0.28	0.20
Insoluble dietary fiber	0.11	0.10	0.27	1.53	0.33	(0.06 - 0.58)		0.22	0.24
Total dietary fiber	0.12	0.12	0.30	1.44	0.36	(0.09 - 0.60)		0.34	0.27

Median	0.34	0.31	0.38	1.36	0.45			0.46	0.35
Average	0.33	0.32	0.38	1.54	0.46			0.29	0.37

* : For n=73, r > 0.24 (p<0.05), r > 0.31 ( p<0.01), r > 0.39 (p<0.001).

† : All energy and nutrients intakes were log_e_- transformed to improve normality.

‡ : Energy intake was adjusted using residual model.

§ : Ratio of within-person to between-person variance of nutrient intakes from three-day weighed diet records.

| : De-attenuated correlation coefficient is calculated using ratio of within- to between-person variation measured with three-day weighed diet records.

¶ : De-attenuation only.

CI: confidence interval.

De-attenuated, log-transformed and energy-adjusted Pearson’s CCs between intakes of nutrients quantified with the FFQ and 3d-WDRs were distributed from 0.10 (vitamin B1) - 0.38 (carotene and folate) - 0.66 (carbohydrate energy %) for females (Table 3), and energy-adjusted Spearman’s rank CCs were distributed from 0.11 (vitamin B_1_) - 0.34 (protein energy % and SFAs) - 0.47 (calcium).

**Table 3.** Pearson’s and Spearman’s rank correlation coefficients between intakes of energy and 26 nutrients measured with three-day weighed diet records and food frequency questionnaire for females.

Nutrient	Pearson’s CCs*							Spearman’s rank CCs*	
	
	Crude	Log- transformed^†^	Log- transformed and energy- adjusted^‡^	*σ*^2^w/*σ*^2^b^§^	De-attenuated, log-transformed and energy-adjusted^|^ (95% CI)			Crude	Energy-adjusted
Energy	0.38	0.38		0.97	0.44	(0.30 - 0.65)	^¶^	0.37	
Protein	0.31	0.31	0.29	1.60	0.36	(0.25 - 0.62)	**	0.30	0.33
Fat	0.29	0.29	0.40	1.32	0.48	(0.40 - 0.72)		0.22	0.38
Carbohydrate	0.48	0.52	0.55	1.05	0.64	(0.61 - 0.85)		0.45	0.44

Protein energy%	0.33	0.33	0.30	1.61	0.37	(0.26 - 0.63)		0.37	0.34
Fat energy%	0.36	0.37	0.40	1.33	0.48	(0.40 - 0.72)		0.33	0.37
Carbohydrate energy%	0.55	0.57	0.57	1.07	0.66	(0.64 - 0.87)		0.45	0.46

Saturated fatty acids	0.40	0.39	0.35	1.33	0.42	(0.32 - 0.66)		0.35	0.34
Monounsaturated fatty acids	0.21	0.18	0.28	1.54	0.34	(0.22 - 0.60)		0.12	0.26
Polyunsaturated fatty acids	0.09	0.13	0.20	1.73	0.25	(0.10 - 0.51)		0.05	0.16
n-6 Polyunsaturated fatty acids	0.11	0.16	0.25	1.60	0.31	(0.14 - 0.46)		0.20	0.22
n-3 Polyunsaturated fatty acids	0.09	0.12	0.17	2.50	0.23	(0.06 - 0.39)		0.17	0.17
n-3 Highly-unsaturated fatty acids	0.17	0.27	0.27	2.10	0.35	(0.19 - 0.49)		0.29	0.27
Cholesterol	0.13	0.15	0.14	2.42	0.19	(0.02 - 0.47)		0.15	0.17

Calcium	0.48	0.52	0.52	0.85	0.59	(0.53 - 0.78)		0.50	0.47
Iron	0.31	0.33	0.38	1.03	0.44	(0.34 - 0.66)		0.33	0.37

Carotene	0.28	0.28	0.30	1.69	0.38	(0.28 - 0.65)		0.31	0.30
Vitamin A	0.11	0.14	0.17	1.90	0.22	(0.06 - 0.48)		0.22	0.24
Vitamin D	0.18	0.27	0.29	2.64	0.40	(0.33 - 0.73)		0.25	0.26
Vitamin E	0.03	0.03	0.14	1.63	0.17	(0.00 - 0.41)		0.00	0.14
Vitamin B_1_	0.11	0.09	0.08	2.12	0.10	(-0.10 - 0.35)		0.13	0.11
Vitamin B_2_	0.42	0.37	0.37	1.05	0.43	(0.32 - 0.65)		0.38	0.38
Folate	0.25	0.27	0.34	0.84	0.38	(0.25 - 0.59)		0.29	0.36
Vitamin C	0.40	0.40	0.46	0.79	0.52	(0.43 - 0.71)		0.43	0.43

Soluble dietary fiber	0.23	0.26	0.31	1.37	0.37	(0.25 - 0.62)		0.28	0.36
Insoluble dietary fiber	0.31	0.30	0.37	1.35	0.46	(0.36 - 0.70)		0.32	0.37
Total dietary fiber	0.33	0.34	0.40	1.23	0.47	(0.38 - 0.71)		0.34	0.41

Median	0.29	0.29	0.31	1.37	0.38			0.30	0.34
Average	0.27	0.29	0.32	1.51	0.39			0.28	0.31

*: For n=129, r > 0.20 (p<0.05), r > 0.26 (p<0.01), r > 0.32 (p<0.001).

† : All energy and nutrients intakes were log_e_- transformed to improve normality.

‡ : Energy intake was adjusted using residual model.

§ : Ratio of within-person to between-person variance of nutrient intakes from three-day weighed diet records.

| : De-attenuated correlation coefficient is calculated using ratio of within- to between-person variation measured with three-day weighed diet records.

¶ : De-attenuation only.

CI: confidence interval.

Median percentages of exact agreement, agreement within adjacent categories, and disagreement according to the quartile classification of energy-adjusted nutrient intakes quantified with the FFQ and 3d-WDRs were 33, 74, and 5 for males (Table 4), and 35, 76, and 7 for females (Table 5), respectively.

**Table 4.** Comparison of nutrient intakes between three-day weighed diet records and food frequency questionnaire according to quartile classification for males.

Nutrient	Crude (%)			Energy-adjusted (%)		
	
	Exact agreement	Agreement within adjacent categories	Disagreement	Exact agreement	Agreement within adjacent categories	Disagreement
Energy	33	74	3			
Protein	33	66	8	29	75	5
Fat	32	75	8	42	84	3
Carbohydrate	41	85	0	42	92	0

Protein energy%	29	75	5	32	77	4
Fat energy%	45	79	4	41	79	3
Carbohydrate energy%	51	89	3	49	93	0

Saturated fatty acids	30	75	8	41	85	5
Monounsaturated fatty acids	33	73	7	29	71	4
Polyunsaturated fatty acids	27	71	5	32	74	7
n-6 Polyunsaturated fatty acids	29	71	11	26	62	17
n-3 Polyunsaturated fatty acids	25	74	12	28	71	15
n-3 Highly-unsaturated fatty acids	31	74	6	33	70	9
Cholesterol	32	70	4	25	70	12

Calcium	30	77	5	32	78	3
Iron	30	68	5	42	82	4

Carotene	32	68	10	37	66	10
Vitamin A	29	60	11	27	66	8
Vitamin D	37	74	4	38	75	7
Vitamin E	26	66	11	29	71	7
Vitamin B_1_	23	66	7	36	66	5
Vitamin B_2_	29	78	3	42	82	1
Folate	30	73	7	38	79	5
Vitamin C	33	67	5	33	74	3

Soluble dietary fiber	23	56	12	32	68	12
Insoluble dietary fiber	22	62	11	33	70	7
Total dietary fiber	26	62	10	26	70	5

Median	30	73	7	33	74	5
Average	31	71	7	34	75	6

**Table 5.** Comparison of nutrient intakes between three-day weighed diet records and food frequency questionnaire according to quartile classification for females.

Nutrient	Crude (%)			Energy-adjusted (%)		
	
	Exact agreement	Agreement within adjacent categories	Disagreement	Exact agreement	Agreement within adjacent categories	Disagreement
Energy	31	75	5	33	77	5
Protein	36	73	7	34	75	4
Fat	36	68	9	36	76	6
Carbohydrate	40	76	5	41	78	5

Protein energy%	35	78	5	35	77	3
Fat energy%	33	73	8	37	74	7
Carbohydrate energy%	40	78	5	40	81	5

Saturated fatty acids	33	74	6	39	79	9
Monounsaturated fatty acids	33	68	12	36	72	8
Polyunsaturated fatty acids	26	64	13	27	68	11
n-6 Polyunsaturated fatty acids	29	71	11	26	62	13
n-3 Polyunsaturated fatty acids	25	74	12	28	71	12
n-3 Highly-unsaturated fatty acids	31	74	6	33	70	7
Cholesterol	31	66	11	33	73	12

Calcium	38	81	5	36	83	5
Iron	33	72	7	35	77	5

Carotene	32	77	8	33	73	6
Vitamin A	29	68	6	33	73	9
Vitamin D	32	74	9	29	74	9
Vitamin E	22	63	14	26	67	9
Vitamin B_1_	30	67	10	29	65	9
Vitamin B_2_	35	76	6	35	75	5
Folate	32	74	9	40	74	7
Vitamin C	39	78	3	36	78	4

Soluble dietary fiber	33	72	5	29	76	4
Insoluble dietary fiber	39	74	9	40	77	5
Total dietary fiber	40	73	7	40	76	5

Median	33	73	7	35	76	6
Average	33	73	8	34	75	7

## DISCUSSION

Because our FFQ is brief, covering 47 foods/food groups, the mean daily intakes of energy and 26 macro- and micro-nutrients determined with the FFQ were, as expected, generally smaller than those measured with the 3d-WDRs.^19^^-^^21^ De-attenuated, log-transformed and energy-adjusted Pearson’s CCs between intakes of selected nutrients quantified with the FFQ and 3d-WDRs were distributed from 0.10 - 0.86 and energy-adjusted Spearman’s rank CCs were from 0.11 to 0.76. For most nutrients, fairly high relative validity values for the FFQ were achieved with reference to the 3d-WDRs. But the disagreement values for certain nutrients were not negligible and non-differential misclassification will unduly underestimate the risk.^22^ Our FFQ thus should be deliberately applicable to rank individuals according to consumption of energy and nutrients selected for dietary studies in middle-aged Japanese.

Relative validity values are dependent on various parameters, such as person, place, time, and study protocols, which include the study subjects (e.g., people in the general population vs. dietitians/nurses), study devices adopted, interval between the two batteries of tests studied, sequence of the batteries, number of food items in the FFQ, procedures and days of DRs, and diversity of food intake (e.g. Japanese, Chinese and American diets).^2^^-^^4^

Relative validity values for macronutrients and respective energy % were reasonably high, but those for some micronutrients, including cholesterol, vitamins, minerals and dietary fibers, were rather low because the two methods measured different profiles of dietary consumption. The former inquired about dietary habits during the preceding year, and the latter surveyed actual food intakes for 3 days. WDRs are accurate without recall bias, but do not necessarily indicate habitual food consumption. Naturally, the two values do not necessarily correlate well with each other. It is also well known that great intra-individual variation exists by day, week and season for micronutrients, including vitamins and minerals.^16^^-^^19^^,^^23^ Three days are not long enough to assess the actual consumption of those nutrients and relative validity indices are invariably low, particularly for nutrients with high within-individual variation. Thus, short-day WDRs cannot be accepted as the gold standard. Furthermore, the both values estimated with FFQ and 3d-WDRs appear underestimated partly because incompleteness of the database published.^9^^-^^12^ Accordingly, our investigation should rightly be called a “*relative*” validation study, and the indices need to be carefully evaluated.

An FFQ covering 47 foods/food groups may not be adequate for accurately assessing consumption of energy and 26 macro- and micro-nutrients. We formerly developed an SQFFQ with 118 foods/food groups. Its relative validity indices against 28d-WDRs (consecutive 7 day-WDRs x 4 seasons) were more favorable than with the short FFQ,^6^ which may be partly explained by the number of included foods/food groups. In general, the greater the number of foods/food groups listed in the FFQ, the higher the relative validity values, but the lower the compliance among study subjects.^2^^-^^4^ In addition, the fact that portion/serving size is requested by the SQFFQ, but not by the FFQ, except for staple foods, may be another reason for variation in the relative validity indices.

Because our long SQFFQ was applied to Japanese dietitians,^6^ it is also plausible that the relative validity indices were more favorable than with subjects from the general populace. Reducing the study subjects’ burden appears critical and questionnaires should be designed to be reasonably short when self-administered by the general public, especially for large-scale epidemiological studies. We thus had to shorten our questionnaire to maintain high compliance and still be able to rank the study subjects according to their nutrient intakes.

The sequence of application of study devices also appears crucial,^2^^-^^4^ The FFQ should be first administered and relative validity figures then evaluated with DRs/WDRs distributed later because FFQs are delivered to the study subjects in the actual dietary epidemiology settings. With the reverse order, DRs/WDRs invariably yield education/memory effects, by which relative validity values are artificially improved, particularly when the interval between the two batteries of tests is short.

Here, we compared the relative validity values for a short FFQ with less than 100 food items applied to the Japanese general populace. Egami et al.^24^ earlier administered a 97-item FFQ before WDRs (Table 6) and their relative validity indices were almost equivalent to those of our questionnaire, with values for macronutrients also consistently greater than those for micronutrients, including vitamins and minerals. Other DRs/WDRs were delivered prior to respective FFQs,^25^^-^^28^ but as discussed earlier, the figures should be carefully interpreted. The relative validity values for most nutrients in our questionnaire nonetheless stand comparison not only with Japanese data but also with those for brief FFQs employed elsewhere in the world.^19^^,^^21^

**Table 6.** Comparison of validity indices for selected nutrients of Japanese short food frequency questionnaires vs. diet records.

	Takatsuka et al. (1997)*		Egami et al. (1999)^†^			Tsubono et al. (2001)^‡^		Lee et al. (2002)^§^	Ogawa et al. (2003)^|^		Present study (2004)	
No. of food items	31			97		44		36	21		47	
No. of frequency categories	8			9		4 or 6		5 or 6	5		8	
Procedures of dietary records	24 hour-recall x 12 months	4day WDRs x four seasons				7day-WDRs x 4 seasons		7 day WDRs x 4 seasons	7 consecutive day-WDRs		3 day-WDRs	
Sequence of two methods	24H-Rs → FFQ	FFQ_1_ → WDRs		WDRs → FFQ_2_		WDRs → FFQ		WDRs → FFQ	WDRs → FFQ		FFQ → WDRs	

Sex	Male and Female	Male	Female	Male	Female	Male	Female	Male	Male	Female	Male	Female
No. of subjects	31	44	42	44	42	94	107	23	55	58	73	129

Energy	0.55	0.25	0.39	0.21	0.38			0.23	0.55	0.38	0.40	0.44
Protein	0.57	0.19	0.30	0.24	0.53	0.39	0.29	0.44	0.25	0.49	0.50	0.36
Fat	-0.03	0.62	0.30	0.60	0.50	0.55	0.37	0.19	0.37	0.50	0.62	0.48
Carbohydrate	0.34	0.52	0.24	0.46	0.53	0.66	0.29	0.45	0.57	0.43	0.86	0.64

Saturated fatty acids	0.51	0.76	0.37	0.73	0.48						0.64	0.42
Monounsaturated fatty acids	0.12	0.61	0.28	0.63	0.53						0.43	0.34
Polyunsaturated fatty acids	-0.15	0.39	0.42	0.39	0.49						0.44	0.25
Cholesterol	0.52	0.53	0.21	0.50	0.35						0.13	0.19

Calcium	0.69	0.61	0.73	0.71	0.78	0.73	0.57	0.52	0.57	0.67	0.49	0.59
Iron		0.22	0.57	0.12	0.52	0.72	0.48	0.31	0.35	0.47	0.58	0.44

Carotene	0.45	0.36	0.51	0.33	0.46	0.52	0.65		0.57	0.45	0.39	0.38
Vitamin A	0.22	0.46	0.52	0.49	0.45			0.19			0.27	0.22
Vitamin D	0.53										0.65	0.40
Vitamin E	0.50	0.50	0.40	0.58	0.41						0.31	0.17
Vitamin B_1_						0.44	0.41		0.33	0.31	0.26	0.10
Vitamin B_2_						0.60	0.63		0.43	0.54	0.57	0.43
Vitamin C	0.44	0.45	0.40	0.55	0.53	0.49	0.39	0.35	0.58	0.43	0.45	0.52

Soluble dietary fiber								0.52			0.25	0.25
Insoluble dietary fiber								0.56			0.33	0.33
Total dietary fiber		0.45	0.61	0.51	0.64			0.56			0.36	0.36

Median	0.48	0.46	0.40	0.50	0.50	0.55	0.41	0.44	0.49	0.46	0.44	0.37
Average	0.38	0.46	0.42	0.47	0.51	0.57	0.45	0.39	0.46	0.47	0.45	0.37

* : Energy-adjusted Pearson’s correlation coefficient of nutrient intakes, except for energy, calculated with FFQ and DRs.^25^

† : De-attenuated, log-transformed and energy-adjusted Pearson’s correlation coefficient of nutrient intakes, except for energy, measured with FFQ and DRs.^24^

‡ : De-attenuated and energy-adjusted Pearson’s correlation coefficient of nutrient intakes, except for energy, calculated with FFQ and DRs.^26^

§ : Energy-adjusted Pearson’s correlation coefficient of nutrient intakes, except for energy, computed with FFQ and DRs.^27^

| : De-attenuated, age- and energy-adjusted Spearman’s rank correlation coefficients of nutrient intakes, except for energy, measured with FFQ and DRs.^28^

WDR: weighed diet record, FFQ: food frequency questionnaire, DR: diet record.

In conclusion, relative validity values were rather low for several nutrients, but satisfactorily high figures were obtained with most nutrients for our FFQ against the 3d-WDRs values. The questionnaire thus seems applicable to rank individuals according to consumption of energy and nutrients selected in dietary studies in the middle-aged Japanese. Bearing in mind these strengths and weaknesses of our FFQ, it can be administered to the general populace, with caution, to investigate possible associations between dietary intake and disease/health in case-control and cohort studies.
